# YB1 dephosphorylation attenuates atherosclerosis by promoting CCL2 mRNA decay

**DOI:** 10.3389/fcvm.2022.945557

**Published:** 2022-08-04

**Authors:** Yaqin Tang, Zhiwei Li, Hongqin Yang, Yang Yang, Chi Geng, Bin Liu, Tiantian Zhang, Siyang Liu, Yunfei Xue, Hongkai Zhang, Jing Wang, Hongmei Zhao

**Affiliations:** ^1^State Key Laboratory of Medical Molecular Biology, Department of Pathophysiology, Peking Union Medical College, Institute of Basic Medical Sciences, Chinese Academy of Medical Sciences, Beijing, China; ^2^Jilin Zhongtai Biotechnology Co., Ltd, Jilin, China; ^3^The Pathology Department, Beijing Hospital of Traditional Chinese Medicine, The Capital Medical University, Beijing, China

**Keywords:** atherosclerosis, RNA binding protein, phosphorylation, inflammation, mRNA decay

## Abstract

Chronic inflammation is a key pathological process in atherosclerosis. RNA binding proteins (RBPs) have been reported to play an important role in atherosclerotic plaque formation, and they could regulate the expression of inflammatory factors by phosphorylation modification. Y-box binding protein 1 (YB1) is an RBP that has participated in many inflammatory diseases. Here, we found an increased expression of phosphorylated YB1 (pYB1) in atherosclerotic plaques and demonstrated that YB1 dephosphorylation reduced lipid accumulation and lesion area in the aorta *in vivo*. Additionally, we found that inflammatory cytokines were downregulated in the presence of YB1 dephosphorylation, particularly CCL2, which participates in the pathogenesis of atherosclerosis. Furthermore, we demonstrated that *CCL2* mRNA rapid degradation was mediated by the glucocorticoid receptor-mediated mRNA decay (GMD) process during YB1 dephosphorylation, which resulted in the downregulation of *CCL2* expression. In conclusion, YB1 phosphorylation affects the development of atherosclerosis through modulating inflammation, and targeting YB1 phosphorylation could be a potential strategy for the treatment of atherosclerosis by anti-inflammation.

## Introduction

Atherosclerosis is a chronic inflammatory and age-related disease characterized by progressive luminal narrowing and obliteration (1). As reported by the Global Burden of Disease Study 2019, atherosclerotic cardiovascular disease (ASCVD) is a leading cause of death worldwide since 1990 (2). Although lipid-lowering and antithrombotic agents are currently applied for clinical therapy in patients with atherosclerosis, there are still 70–80% residual risks of major adverse cardiovascular events (MACE) among them and are suggested to be associated with insufficient control of anti-inflammatory responses (3).

Currently, statins are the only therapeutic agent which has an anti-inflammatory effect for atherosclerosis in clinical therapy (4, 5), but their anti-inflammatory effect is limited due to the first-pass metabolism and low bioavailability. Beyond that, canakinumab is a monoclonal antibody that targets IL-1β, leading to a substantially lower rate of MACE in patients with post-myocardial infarction (6). Nonetheless, it causes immunosuppression in patients and results in persistent infection. Considering the limitations of anti-inflammation through directly targeting one of the cytokines, new anti-inflammation strategies were required. The transcriptional level of inflammatory genes was controlled by regulatory elements through post-transcription regulation. Focusing on inflammatory mRNA regulatory elements may be a new strategy for atherosclerosis therapy.

RBPs regulate inflammatory cytokine mRNA expression by adjusting their own phosphorylation modification (7) followed by interaction with cis-regulatory elements of cytokines. They have been reported to participate in the progression of chronic inflammatory diseases (8), including cardiovascular diseases (CVDs). Especially in atherosclerosis, where RBPs can modulate the vascular cellular phenotype (9, 10). YB1 (encoded by *YBX1*), as a member of RBPs, regulates multiple biological processes, including cell apoptosis, differentiation, proliferation, and stress response (11–13), and it also involves a variety of DNA/RNA-dependent events, including DNA repair, transcription, mRNA splicing, mRNA stability, and translation (14, 15). Additionally, YB1 phosphorylation is a post-translational modification that plays an important role in the progression of inflammation (16–19), but the mechanism of pYB1 in regulating inflammation is still unclear and warrants further exploration.

Vascular smooth muscle cells (VSMCs) are among the critical cell types involved in the pathogenesis of atherosclerosis. In addition, the major source of extracellular matrix and plaque cells originated from VSMCs and VSMC-derived cells. VSMCs secrete pro-inflammatory factors and contribute to many plaque cell phenotypes, including foam cells and macrophage-like cells, they are present at all stages of the development of atherosclerosis (20). A single-cell RNA sequencing (scRNA-seq) study revealed that YB1 is a master regulating factor for VSMC phenotype switch which contributes to atherosclerotic plaque formation (21). These studies indicated the important role of VSMCs in atherosclerosis, while the role played by pYB1 in VSMCs in atherosclerosis remains unknown.

To further explore and confirm the roles of pYB1 in atherosclerosis, we detected the expression of pYB1 and YB1 in human and mouse atherosclerotic lesions. Subsequently, gain and loss of function experiments were performed *in vivo*, and the function of YB1 phosphorylation at Ser-100 was also validated *in vivo*. Finally, the molecular mechanism of pYB1 in atherosclerosis was explored *in vitro*, we constructed the YB1 phosphorylation site (Ser-100) mutant stable smooth muscle cell line (3ds-V5) and performed RNA profiling through RNA-seq. The candidate genes regulated by pYB1 were further verified and the regulation mechanism was investigated by RNA immunoprecipitation and mRNA decay assay.

In this study, we provide evidence that pYB1 aggravates atherosclerosis by regulating inflammatory mRNA expression. Herein, the dephosphorylation of YB1 at Ser-100 may become a potential target for atherosclerotic therapy.

## Materials and methods

### Analysis of the bulk transcriptomics data

All bioinformatics analysis was performed with R software (v3.6.0). DEGs between atherosclerosis (carotid plaque tissues) and controls (intact carotid tissues) from the dataset GSE43292 were obtained using the limma package (v3.42.2) (22). DEGs with adjusted *p* <0.05 were selected for further analysis. DEGs between human stable and unstable atherosclerotic plaques from the dataset GSE120521 were obtained using the limma package with *p* < 0.05, and absolute fold change >1.2. The Human RBP list was downloaded from the RBPDB database (http://rbpdb.ccbr.utoronto.ca/). Next, we identified RBPs responsible for atherosclerosis from the intersection of DEGs and the human RBPs list. The Venn diagram was plotted using the VennDiagram package (v1.6.20). The heatmap was plotted using the ComplextHeatmap package (v2.2.0).

### Analysis of single-cell RNA sequencing data

Data were analyzed using the Seurat package (v3.2.3). Human and mouse scRNA-seq data from the dataset GSE131780 and the data were analyzed separately using the same pipeline: Data of each sample was normalized by the number of UMIs per cell and 4,000 variable features were analyzed. Then, the data from all the samples were integrated using canonical correlation analysis (23), and unsupervised clustering was performed following the standard procedure of Seurat. Cell clusters were annotated using reported cell type markers (24).

### Analysis of RNA-seq data

Briefly, the reads of the RNA-seq data of 3ds-V5 and YB1-V5 were aligned to the Rattus_norvegicus reference genome (v6.0.92) using HISAT2 (v2.1.0) software (25), DEGs between the 3ds-V5 and YB1-V5 (control) group were got using the DESeq2 package (v1.26.0) (26). DEGs that meet the following criteria were selected: adjusted *p* < 0.01, and absolute fold change > 2. Gene Ontology (GO) enrichment analysis was performed using the Metascape webtool (v3.14.3) (27) and plotted using ggplot2 (v3.3.3). The heatmap was plotted using the pheatmap package (v1.0.12).

### Cell culture

The Rat Aortic Smooth Muscle Cell was purchased from ScienCell Research Laboratories, USA and cultured in Smooth Muscle Cell Medium (SMCM, Sciencell) containing 2% fetal bovine serum (FBS, Gibco), 1% smooth muscle cell growth supplement (SMCGS, Sciencell), and 1% penicillin/streptomycin solution (Sciencell). HEK293T Cell was purchased from the National Infrastructure of Cell Line Resource, China, and cultured in Dulbecco's Modified Eagle Medium (DMEM, Gibco, USA), containing 10% FBS, 100 IU/ml penicillin, and 100 μg/ml streptomycin (Gibco), and incubated in a humidified incubator at 37°C with 5% CO_2_.

### Lentiviral vector construction and virus packaging

Lentiviral vector construction and virus packaging: the mutant mouse YB1 phosphorylation at Ser-100 and YB1 overexpression plasmids were cloned into the *Sgf* I-*Mlu*I sites of the lentiviral vector pLenti-C-Myc-DDK (OriGene Technologies, Maryland, USA) to generate Lv-S100A and Lv-YB1, respectively. YB1 knockdown plasmid was cloned into the *BamH* I-*EcoR* I sites of the lentiviral vector pSIH-H1-siLuc-CopGFP. All the lentiviral vector constructs were verified by DNA sequencing. Virus packing was performed according to the manufacturer's instructions for the lentivirus packaging kit (System Biosciences, CA, USA). Virus particles were concentrated by PEG-it Virus Precipitation Solution (System Biosciences, CA, USA).

Rat mutant vector construction: YB1 (Gene Bank accession no. NM031563) cDNA was cloned between *Hind* III and *Xba* I of the multiple cloning sites in pBluescript II KS (+). All the site-directed YB-1 mutations were generated by PCR and subcloned into pcDNA3.1/V5-His A between *Hind* III and *Xba* I. The constructs were verified by DNA sequencing.

### Mutant cell lines

RASMC at 40% confluence were transfected with 1 μg construct DNA linearized with Pvu I. Around 24 h after transfection, Lipofectamine 2000 (Invitrogen, USA) was removed, and neomycin was added at a concentration of 300 μg/ml. Two weeks after transfection, five colonies from each transfection were isolated. The stably transfected cells were cultured without Neomycin for all experiments.

### Human vascular tissue samples

Human atherosclerotic aortic tissues were obtained from patients undergoing carotid endarterectomy (CEA). Normal aortic tissues were obtained from patients undergoing coronary bypass surgery or organ donors.

### Animals

A total of 27 male *ApoE*^−/−^ (C57BL/6J) mice aged 8 weeks old were purchased from Vital River Laboratory Animal Technology Co. Ltd. (Beijing, China), and maintained at a constant temperature of 23°C and humidity of 55% under specific-pathogen-free (SPF) conditions. Mice were fed a high-fat diet (HFD) until sacrifice. At the age of 12 weeks, mice were randomly divided into five groups accompanied by lentiviral infection *via* tail vein injection. Mice from Lv-control group (*n* = 5) and Lv-GFP (*n* = 5) were treated as empty vector expression controls, mice from Lv-YB1 group (*n* = 6) and Lv-S100A group (*n* = 6) were treated as YB1 over-expression and phosphorylation site mutant (Ser-100), mice from Lv-shYB1 group (*n* = 5) were treated as YB1 knockdown.

### mRNA stability assay

VSMCs were cultured in six-well plates to 60% confluence, then cells were, respectively, transfected with Lv-YB1 and Lv-S100A plasmids using Lipofectamine 2,000 reagent. After 72 h of transfection, cells were treated with 5 μg/ml actinomycin D (Sigma-Aldrich, USA) for the indicated time points.

### RNA preparation and RT-qPCR

Total RNA of cells was isolated by using TRIzol reagent (Invitrogen) according to the manufacturer's instructions. RNA concentration and purity (A260/A280) were assessed using Nanodrop (Thermo Fisher Scientific, USA). The first strand cDNA synthesis was performed with the FastKing RT Kit (TianGen, China) according to the manufacturer's instructions. The RT-qPCR was performed by using a 2x Taq master mix (TianGen, China) as previously described (28). The following primers were used: Rat *CCL2* forward: 5′-TCGGCTGGAGAACTACAAGAGAA-3′, Rat *CCL2* Reverse: 5′-CTTCTGGACCCATTCCTTATTGG-3′; Mouse *YB1* forward: 5′-GGAGAAGTGATGGAGGGTGCT-3′, Mouse *YB1* Reverse: 5′-CCTTCGGAATCGTGGTCTGTA-3′. Final data were calculated by using the 2^−Δ*ΔCt*^ method, and relative gene mRNA expression levels were normalized to that of β*-Actin*.

### Immunoprecipitation

A total of 293T cells were transiently transfected with Lv-control, Lv-YB1, and Lv-S100A plasmids using Lipofectamine 2000 for 48 h to overexpress YB1, and YB1 mutant. After that, cells were lyzed in RIPA (50 mM Tris (PH7.4), 150 mM NaCl, 1mM EDTA, 10% (v/v) glycerol, 1% Triton X-100) for immunoprecipitation. According to the manufacturer's instructions for Anti-Flag-M2 Affinity Gel (Sigma-Aldrich), 800 μl of cell lysate was added to the washed resin (The ratio of suspension to packed gel volume should be 2:1), then the mixed suspension was allowed to shake in a roller shaker gently in 4°C overnight. Next, the supernatant was removed after centrifuging the suspension for 30 s at 5,000 × *g*, and the resin was washed with RIPA three times. Finally, the supernatant was removed and resin-bound proteins were eluted with 0.1 M glycine HCl and separated by sodium dodecyl sulfate-polyacrylamide gel electrophoresis (SDS-PAGE) for subsequent analysis by immunoblotting.

### RNA immunoprecipitation

VSMCs were transiently transfected with Lv-control, Lv-YB1, and Lv-S100A plasmids using Lipofectamine 2,000 for 72 h to overexpress YB1 and YB1 Ser-100 mutant. In addition, cells were treated with PDGF-BB (20 ng/μl) for 3 h. After that, cells were collected and a RIP assay was performed using the RIP assay Kit (MBL Beijing Biotech, China) according to the manufacturer's instructions (29).

### Cytokine assays by ELISA

CCL2 levels in cell culture supernatant or mouse plasma were tested by ELISA (R&D Systems, MN, USA), according to the manufacturer's instructions.

### Western blot analysis

The VSMC cell lysate was prepared in RIPA lysis buffer with a protease inhibitor cocktail for 30 min, and the concentration of total proteins was measured using the BCA Protein Assay Kit (Thermo Scientific). Protein samples were separated on 10% SDS-PAGE and transferred to a polyvinylidene difluoride (PVDF) membrane (Millipore, USA). Next, the membrane was blocked with 5% milk in TBST and incubated with primary antibody (Supplementary Table 1) overnight at 4°C. After washing three times with TBST, the membrane was incubated with a secondary antibody for 1 h at room temperature and then washed with TBST three times. Finally, the membrane was incubated with ECL reagents (Thermo Scientific) for the detection of target bands by the Tanon Chemiluminescence/Fluorescence Image Analysis System (Tanon, China).

### Histological staining and analysis

Fresh tissues (aorta) from humans or mice were embedded in OCT compounds and sectioned with the cryotome (Leica 3050S) at 6 μm/slice. The staining methods for Oil Red O and IHC have been described in the previous study with some modifications (30). Briefly, for IHC, the frozen tissue sections were permeabilized by 0.025% Triton X-100 for 5 min, and endogenous peroxidase activity was quenched with 0.3% H_2_O_2_ for 15 min, then the nonspecific binding was blocked with goat serum for 30 min, the tissue sections were incubated with the primary antibodies (Supplementary Table 2) for overnight at 4°C. After being washed with PBS 3 times, the tissue sections were incubated with anti-rabbit HRP-conjugated secondary antibody for 30 min at room temperature. Finally, the visualization was obtained by using an AEC kit (FuZhou Maixin Biotechnology Development Co., Ltd, 1:200 dilution) for about 15 min. Images were captured using Nikon microscopy. The positive staining areas were analyzed by Image-Pro Plus software.

### Lipid measurement

Blood samples from *ApoE*^−/−^ mice were collected by cardiac puncture and kept on the ice until further centrifugation at 1,500 × *g* for 10 min at 4°C. According to the manufacturer's instructions, the levels of plasma total cholesterol, triglycerides, Low-density lipoprotein cholesterol (LDL-C), and high-density lipoprotein cholesterol (HDL-C) were measured by the total cholesterol assay kit (ApplyGen, Beijing, China), triglycerides assay kit (ApplyGen), LDL-C assay kit (ApplyGen), and HDL-C assay kit (ApplyGen), respectively. Finally, the absorbance values were detected with a microplate spectrophotometer.

### Statistical analysis

Data analysis was performed using GraphPad Prism 8.0 and presented as the mean ± SEM. All the cell experiments were performed for three independent experiments. Histological and IHC stains of animal experiments were assessed by a single observer blinded to treatment, and numbers of animals (*n*) were described in the figure legends. In general, the unpaired Student's *t*-test and the one-way ANOVA analysis with Tukey's post-test were used for evaluating the differences between the two groups and multiple comparisons, respectively. Differences with *p* < 0.05 were considered statistically significant (two-sided).

## Results

### YB1 expression was upregulated in patients with carotid atherosclerosis by transcriptomic profiling

**Figure 1:**
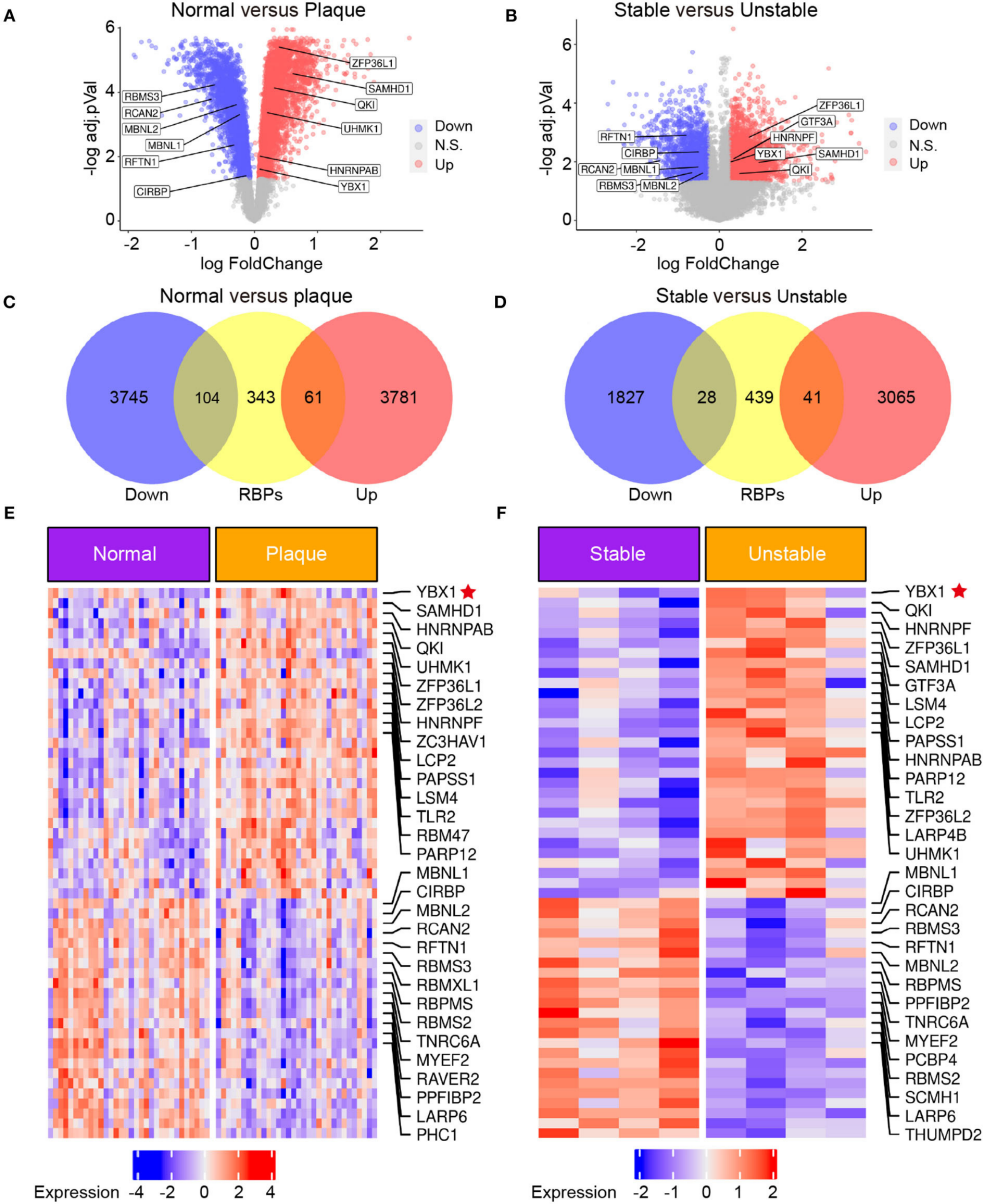
The expression of YB1 was upregulated in patients with carotid atherosclerosis by transcriptomic profiling. **(A,B)** Volcano plot visualization of the differentially expressed genes (DEGs) between normal tissue and atherosclerotic plaque in patients with atherosclerosis [**(A)** GSE43292, *n* = 32] and between stable and unstable plaque in patients with atherosclerosis [**(B)**, GSE120521, *n* = 4], respectively. Red dots indicate upregulated genes in plaque, and blue dots indicate genes with decreased expression; **(C,D)**: Venn diagram indicating the upregulated and downregulated RBPs in the GSE43292 **(C)** and GSE120521 **(D)** datasets, respectively. **(E,F)**: Heatmap illustrates substantially upregulated RBPs in GSE43292 **(E)** and GSE120521 **(F)** datasets, respectively. The expression data was z-scores scaled by rows. Each row represented an RBP and was shown in descending order by average expression.

Several RBPs have been demonstrated to have implications for the initiation and development of CVDs (9). To explore the RBPs profiling in atherosclerosis, the transcriptome microarray data of patients with carotid atherosclerosis were analyzed. We identified 7,691 differentially expressed genes (DEGs) between carotid atheroma and paired macroscopically intact tissue adjacent to the atheroma plaque of each patient (Figure 1A) and 4,961 DEGs between stable and unstable atherosclerotic plaque (Figure 1B). A total of 165 RBPs were substantially changed in carotid atheroma (Figure 1C), and 69 RBPs were differentially expressed in unstable atherosclerotic plaque (Figure 1D). A total of 30 shared up-regulated RBPs of the two data were found through intersection of the DEGs, among the DEGs, YB1 was the most abundantly expressed RBP both in carotid atheroma (Figure 1E) and unstable atherosclerotic plaque (Figure 1F).

### YB1 and phosphorylated YB1 were elevated in atherosclerotic plaques

**Figure 2:**
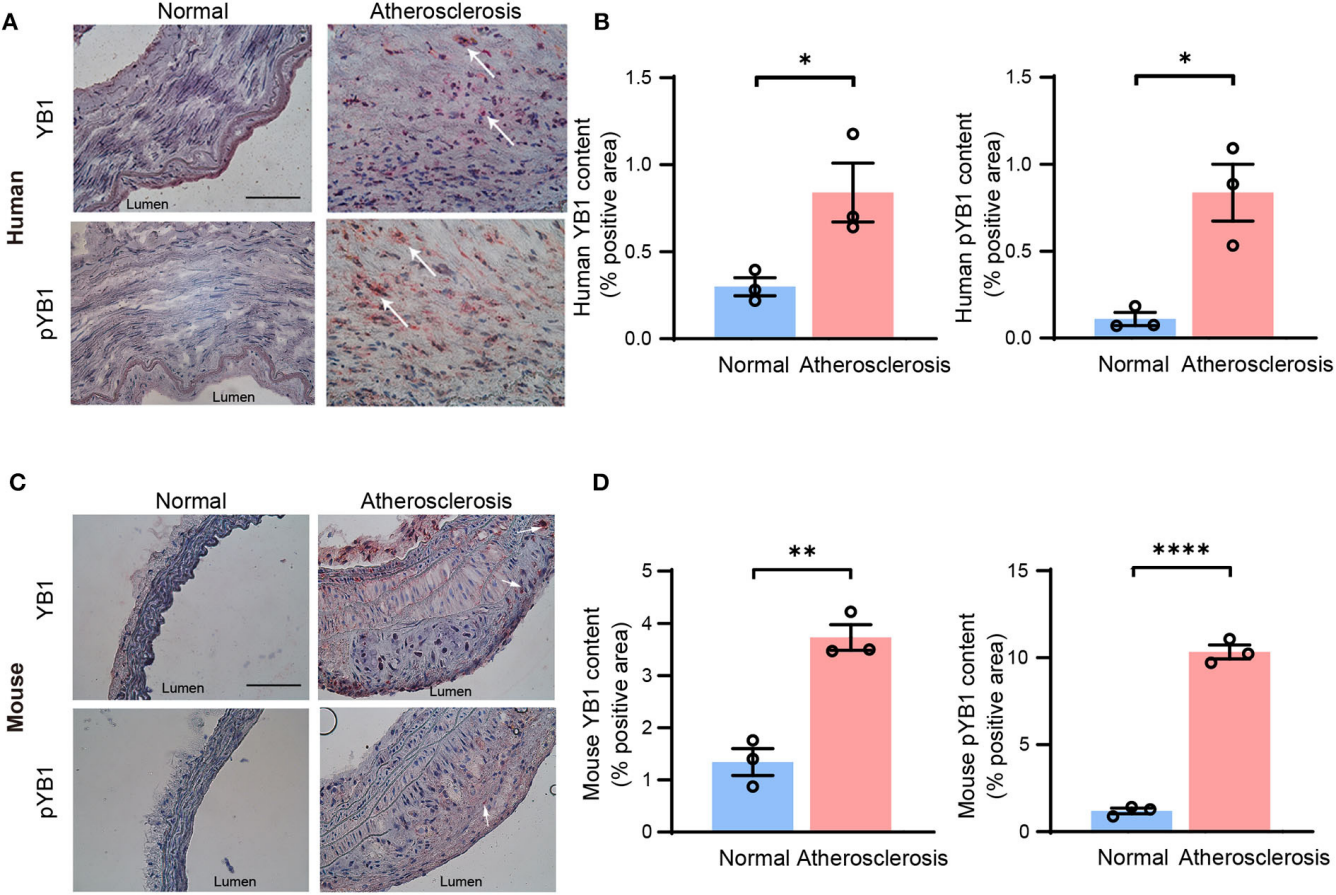
The expression of YB1 and phosphorylated YB1 were elevated in atherosclerosis. **(A)** Representative immunohistochemical staining image of YB1 and pYB1 in the human aorta, and the related quantitative analysis **(B)**. **(C)** Representative immunohistochemical staining images of YB1 and pYB1 in the aortic arch of *ApoE*^−/−^ mice, and the related quantitative analysis **(D)**. Data are presented as the mean ± SEM. Student's two-tailed *t-*test. ^*^P < 0.05, ^**^P < 0.01, ^****^P < 0.0001 vs. Normal group. Scale bar = 100 μm (*n* = 3 for each group).

To confirm our findings, the protein expression levels of YB1 and pYB1 in human and mouse atherosclerotic lesions were detected by immunohistochemical (IHC). The results showed that the expression of pYB1 and YB1 were markedly elevated in human atherosclerotic plaques compared with the vessels of normal tissues by IHC staining analysis (Figures 2A,B). Consistent with the above results in human tissues, pYB1 and YB1 were also elevated in mouse atherosclerotic tissues compared with the normal tissues (Figures 2C,D).

### YB1 dephosphorylation attenuates atherosclerosis in *ApoE^−/−^* mice

**Figure 3:**
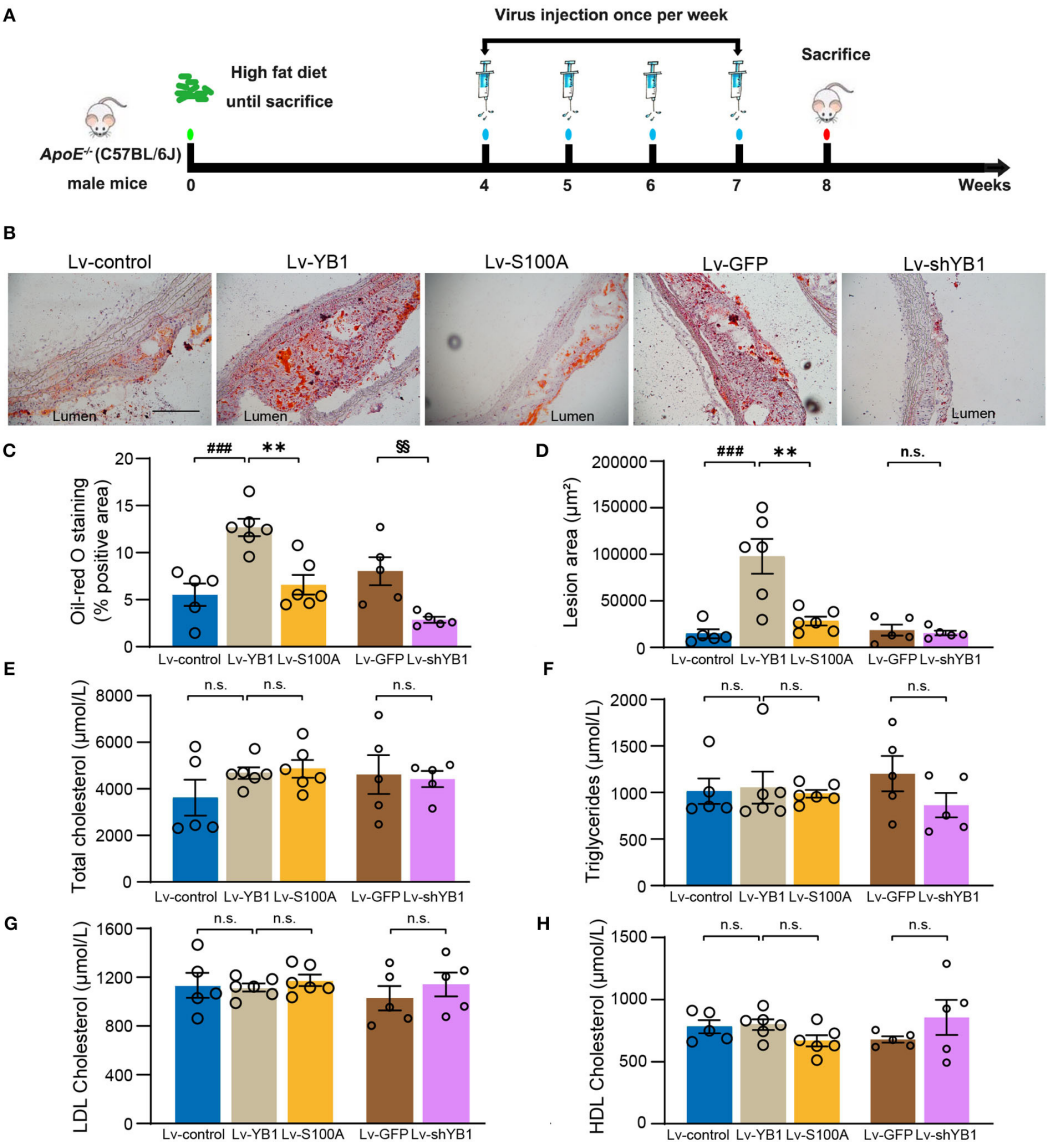
YB1 dephosphorylation attenuates atherosclerosis in *ApoE*^−/−^ mice. **(A)** Schematic diagram of the experimental animal model of atherosclerosis with lentivirus infection. **(B)** Representative images of Oil Red O staining of the aortic arch, and the related quantitative analysis **(C)**. **(D)** Lesion area quantification of the aortic arch. **(E–H)** Serum lipid profiles. Data are presented as the mean ± SEM. One-way ANOVA or Student's two-tailed *t*-test. ^###^*P* < 0.001 vs. Lv-control, ^**^P < 0.01 vs. Lv-YB1, ^§§^*P* < 0.01 vs. Lv-GFP. N.S. indicated the data have no statistical difference. Scale bar = 100 μm (*n* = 5 or 6 for each group).

To investigate the role of pYB1 and YB1 in the development of atherosclerosis, we constructed and packaged YB1 knockdown (Lv-shYB1), over-expressed (Lv-YB1), and Ser-100 phosphorylation site mutant lentivirus (Lv-S100A). LV-control, Lv-shYB1, Lv-YB1, Lv-S100A, and Lv-GFP were respectively injected into *ApoE*^−/−^ mice *via* tail vein one time per week (1 × 10^7^ Pfu per injection per mouse) at the age of 12 weeks. After gain or loss of YB1 function using tail vein injection lentivirus for another 4 weeks, the mice were sacrificed (Figure 3A). Lentivirus-mediated YB1 overexpression elevated pYB1 expression in the aortic tissue compared with the YB1 S100A mutant or the Lv-control group (Supplementary Figures 1A, B). Furthermore, the YB1-knockdown in aortic tissue was confirmed by IHC, showing a considerable decrease (Supplementary Figures 1C, D). Remarkably, analysis of atherosclerotic lesion formation by Oil-Red O staining revealed that lipid accumulation and lesion area increased more than two times after overexpressing YB1 (compared with the control group), while lipid accumulation and lesion area reversed in the YB1 S100A mutant group when compared with the YB1 overexpression group. In addition, the lipid deposition was alleviated obviously after YB1 knockdown both in the aortic arch(Figures 3B–D) and aortic root (Supplementary Figures 1E–H). These indicated that YB1 phosphorylation played a crucial role in atherosclerotic plaque formation.

To explore the effect of pYB1 on lipid dysfunction which was important in atherosclerosis, we detected the plasma lipid levels. As shown in Figures 3E–H, there was no considerable difference in total cholesterol, triglyceride, LDL cholesterol, and HDL cholesterol levels. These results suggested that YB1 dephosphorylation attenuated atherosclerosis was independent of plasma lipid levels.

### Phosphorylated YB1 expression in VSMCs was elevated in atherosclerosis

**Figure 4:**
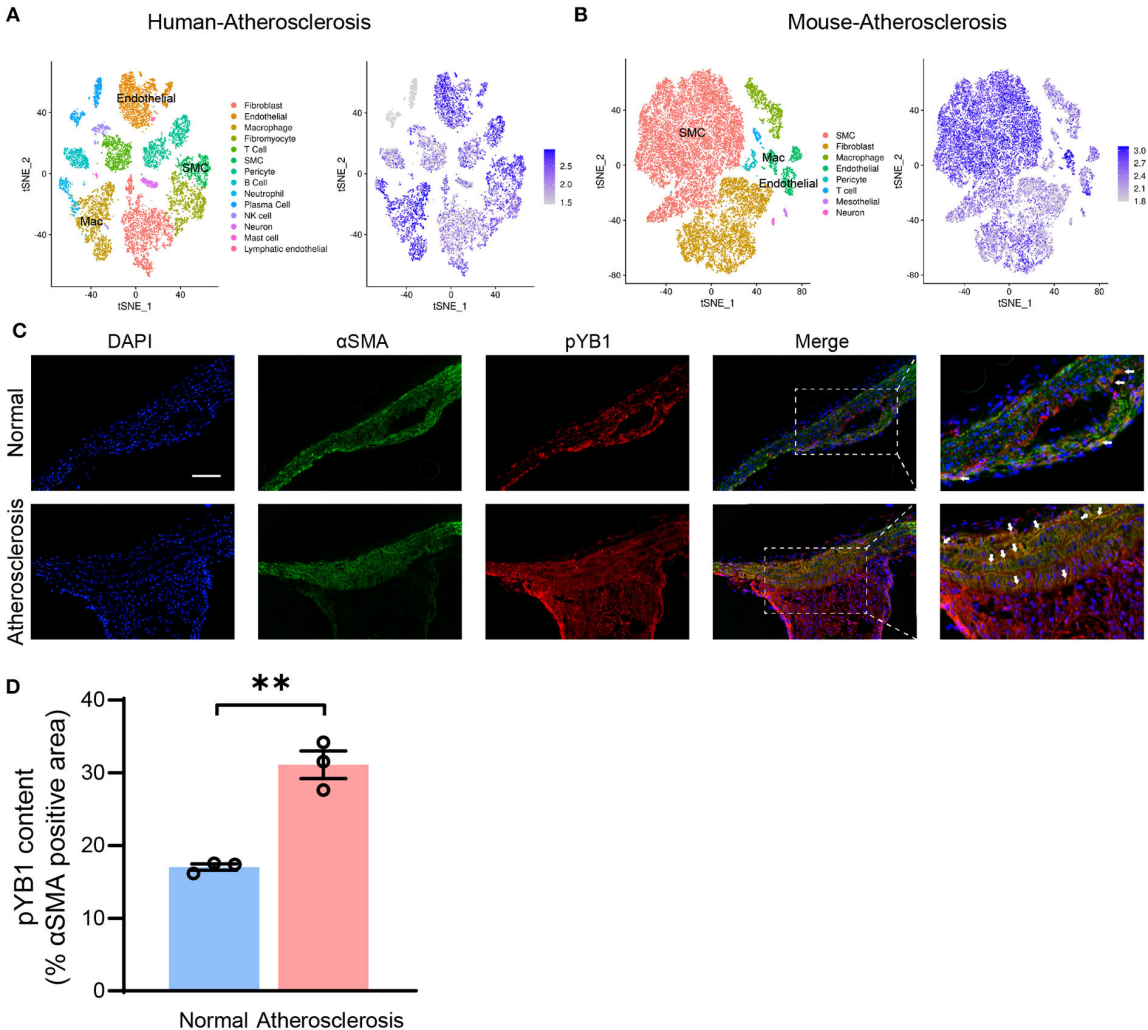
Phosphorylated YB1 was elevated in smooth muscle cells in atherosclerotic mice. **(A)** t-stochastic neighbor embedding (t-SNE) visualization of cell types present in human atherosclerosis aortic tissue (left), and the expression of the gene *YBX1* (encodes YB1, right). **(B)** t-SNE visualization of cell types present in mouse atherosclerosis aortic tissue (left), and the expression of YB1 (right). **(C)** Representative images of immunofluorescent co-staining of αSMA and pYB1 in aortic arches, and the related quantitative analysis **(D)**. Abbreviations in **(A)** SMC, smooth muscle cell; Mac, macrophage. Data are presented as the mean ± SEM. Student's two-tailed *t*-test. ^**^P < 0.01 vs. Normal, Scale bar = 100 μm (*n* = 3–5 for each group).

Accumulating studies highlight that VSMCs play a crucial role in atherosclerosis (31), To further explore the mechanism of the pYB1 in atherosclerosis, we analyzed the cellular localization of YB1 in different types of cells in humans and mouse atherosclerotic tissues by scRNA-seq (24). Cell types were annotated based on the expression patterns of canonical marker genes (Supplementary Figures 2A, B). We found that YB1 was mainly expressed in VSMCs, endothelial cells, and macrophages (Figures 4A,B), and the expression of YB1 was increased in VSMCs, fibroblasts, and macrophages in the mouse aorta at the 8^th^ and 16^th^ weeks of the atherosclerotic model compared to the controls (Supplementary Figures 2C, D). We then validated the localization of pYB1 in mouse aorta and found that pYB1 was primarily expressed in VSMCs, only marginally expressed in the endothelial cells and macrophages (Supplementary Figure 3A). While pYB1 expression in VSMCs was substantially elevated in the atherosclerotic lesion in *ApoE*^−/−^ mice compared with normal ones (Figures 4C,D). Furthermore, immunofluorescence co-staining revealed that Flag or GFP tag colocalized with αSMA in lentivirus mice (Supplementary Figures 3B,C). This indicated that lentivirus successfully infected the aorta and functioned well in VSMCs. Therefore, we further explored the specific mechanism of pYB1 in VSMCs.

### YB1 dephosphorylation promotes glucocorticoid receptor-mediated CCL2 mRNA decay

**Figure 5:**
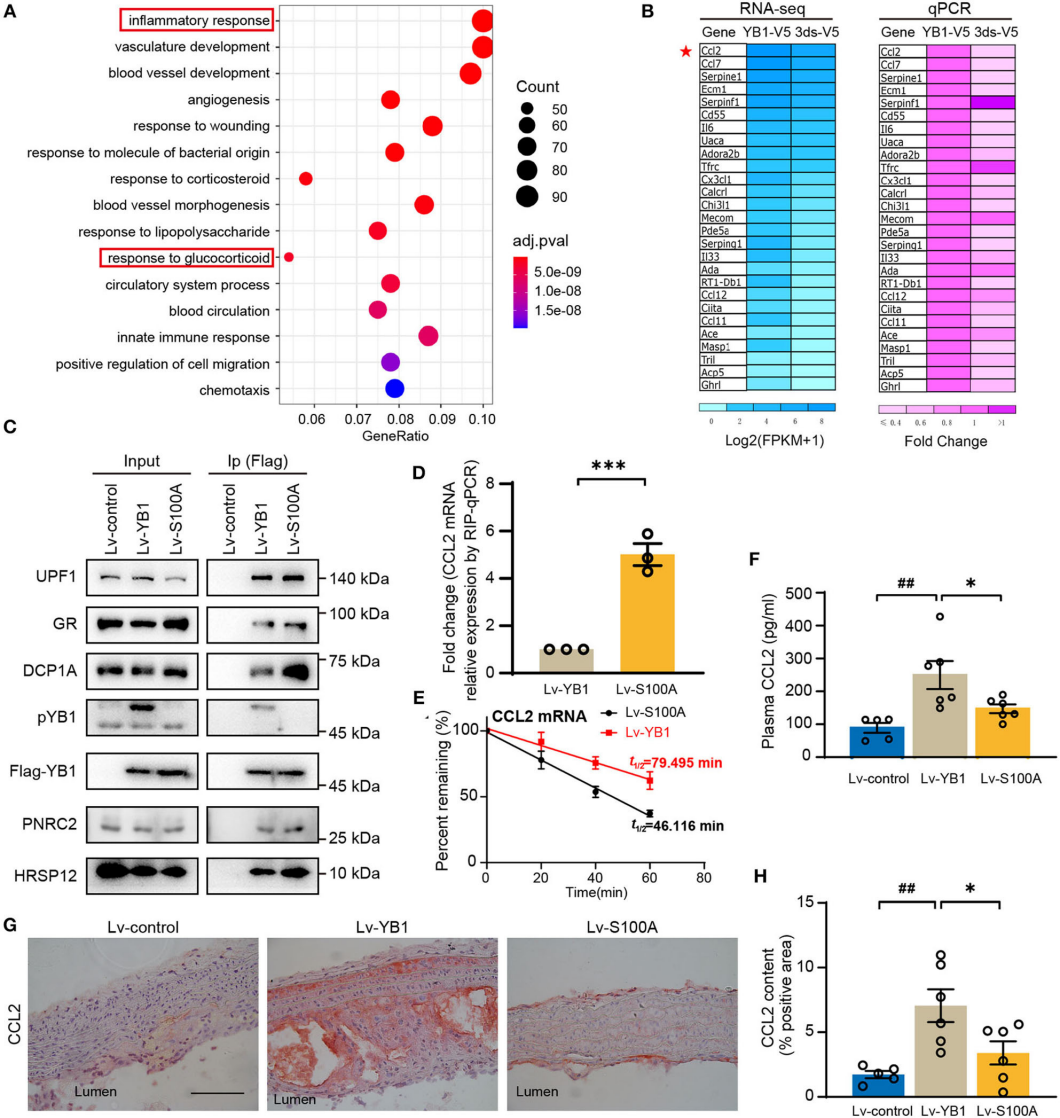
YB1 dephosphorylation decreased CCL2 expression by promoting mRNA decay in SMCs. **(A)** Top 15 enriched Gene Ontology using DEGs between the YB-1-V5 and 3dS-V5 group; **(B)** Heatmap showing the expression of representative inflammatory-related genes in YB-1-V5 and 3dS-V5 group in the RNA-seq data and its validation by RT-qPCR, respectively. **(C)** Flag-tagged YB1 was immunoprecipitated using Flag M2 beads and subjected to the Western blot for the determination of the GMD (glucocorticoid receptor-mediated mRNA decay) complex. **(D)** Flag-tagged YB1 RIP followed by qPCR (RIP-qPCR) analysis between the Lv-YB1 and Lv-S100A group. **(E)** The half-live of CCL2 was decreased in the Lv-S100A group compared with the Lv-YB1 group. **(F)** Plasma CCL2 of *ApoE*^−/−^ mice from Lv-control, Lv-YB1, Lv-S100A group. **(G)** Representative images of immunohistochemical staining of CCL2 in the mouse aortic arch from Lv-control, Lv-YB1, and Lv-S100A groups, and the related quantitative analysis, respectively **(H)**. Data are presented as the mean ± SEM. Statistics **(D)**: Student's two-tailed *t*-test, **(F,H)**: One-way ANOVA. ^##^*P* < 0.01 vs Lv-control, ^*^P < 0.05, ^***^P < 0.001 vs. Lv-YB1. Scale bar = 50 μm (*n* = 3 to 6 for each group).

To investigate the specific role of pYB1 in VSMCs, we constructed rat smooth muscle cell lines of stably expressed 3ds-V5 cells (lacking the phosphorylation site at position 100) and YB1-V5 cells (Supplementary Figure 4A), pYB1 was remarkably decreased in 3dS-V5 compared with YB1-V5 cells (Supplementary Figure 4B). By performing Gene Ontology (GO) biological process enrichment analysis on the DEGs between the two cells revealed by RNA-seq, we showed that the predominantly enriched pathway was the “inflammatory response” (Figure 5A). Moreover, genes associated with the inflammatory pathways were mainly pro-inflammatory chemokines and cytokines, this result was further validated by qPCR (Figure 5B). CCL2 is a chemokine that mediates peripheral blood monocytes recruit into the arterial wall, resulting in neointima formation in atherosclerosis (32–35), and CCL2 deficiency reduces atherosclerotic plaque formation in mice of diverse genetic backgrounds (36, 37). As shown in Figure 5B, *CCL2* was highly expressed in the YB1-V5 group and substantially decreased in the 3dS-V5 YB1 mutant. Meanwhile, the GO enrichment analysis in Figure 5A showed that the “response to glucocorticoid” pathway was enriched, which had been reported to be associated with the rapid degradation of *CCL2* mRNA (38–40). Therefore, we hypothesized that pYB1 regulates mRNA levels of cytokines by glucocorticoid receptor-mediated mRNA decay (GMD). To validate this hypothesis, we transfected HEK293T cells with Lv-control, Lv-YB1, and Lv-S100A and then detected the expression of endogenous GMD complexes (GR, PNRC2, DCP1A, UPF1, HRSP12) after immunoprecipitation. As expected, YB1 dephosphorylation promoted the binding of YB1 with GMD complexes (Figure 5C), resulting in an increase of *CCL2* mRNA in the complexes (Figure 5D), which contributes to *CCL2* mRNA reduction by affecting mRNA stability (Figure 5E). These results demonstrated that YB1 dephosphorylation facilitated *CCL2* mRNA binding to the GMD complexes, resulting in the rapid *CCL2* mRNA decay *in vitro*.

In addition, compared with the Lv-YB1 group, the expression level of CCL2 in mouse plasma (Figure 5F) and aorta (Figures 5G,H) were both decreased in the Lv-S100A group. These results indicated the important role of pYB1 in the regulation of CCL2 *in vivo*.

### MK2206 inhibited the phosphorylation of YB1

**Figure 6:**
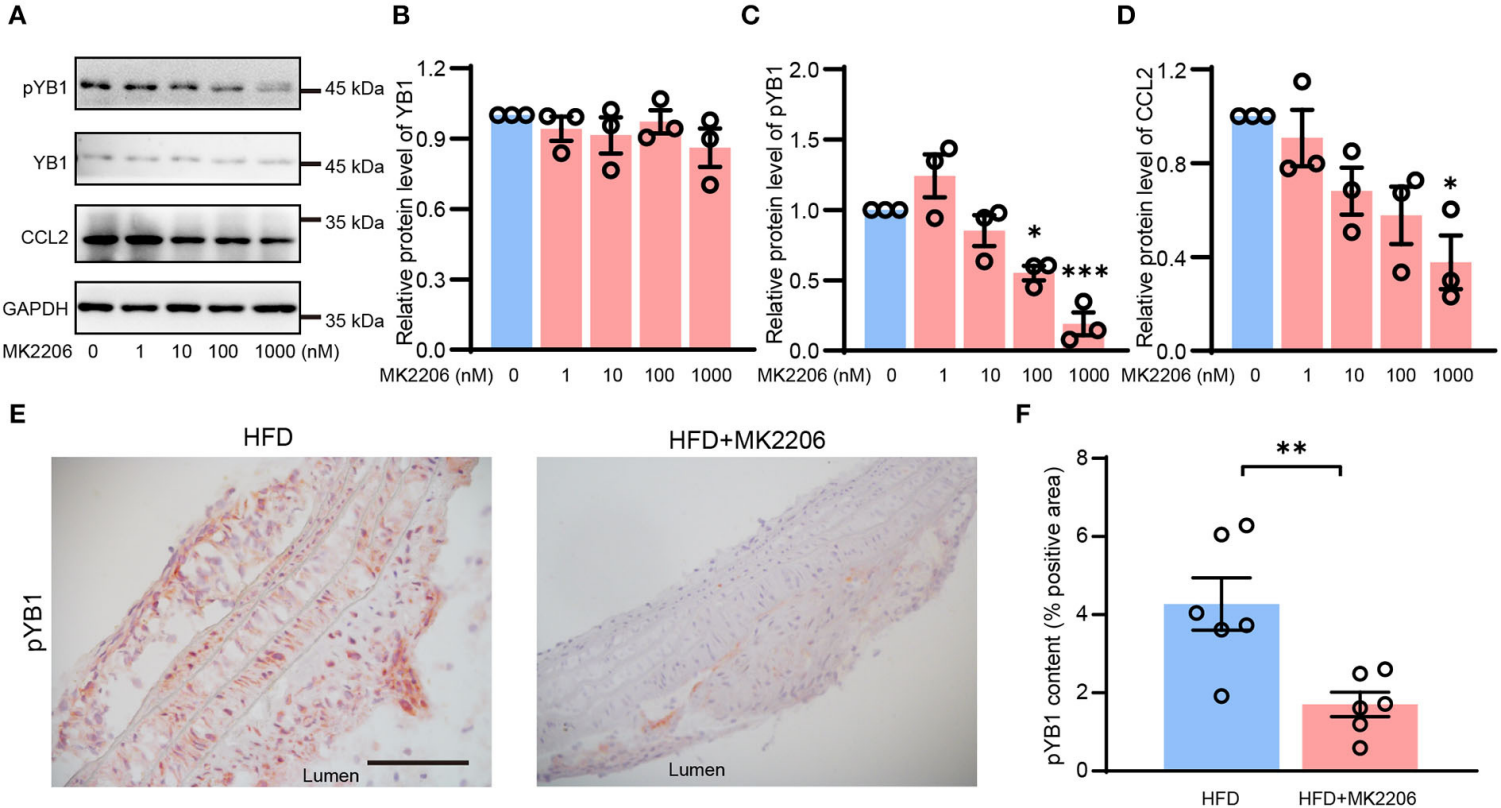
MK2206 inhibited YB1 dephosphorylation and decreased CCL2 expression. Representative immunoblot images **(A)** and quantification of YB1 **(B)**, pYB1 **(C)**, and CCL2 **(D)** in VSMCs treated with MK2206 in a dose-dependent manner for 24 h. **(E)** Representative images of immunohistochemical staining of CCL2 in the mouse aortic arch from HFD and HFD + MK2206 group, and the related quantitative analysis **(F)**. Data are presented as the mean ± SEM. Student's two-tailed *t*-test. ^*^P < 0.05, ^**^P < 0.01, ^***^P < 0.001 vs. 0 nM group or HFD group. Scale bar = 50 μm (*n* = 3 to 6 for each group).

YB1 phosphorylation was regulated by phosphorylated Akt (41), MK2206 is the specific inhibitor of AKT which can inhibit AKT phosphorylation, and it is a drug in clinical phase II. In our previous study (28), we demonstrated that MK2206 suppressed atherosclerosis by inhibiting inflammation, and CCL2 was reduced in this mouse model after the treatment of MK2206. Thus, we speculate that MK2206 may attenuate atherosclerosis by inhibiting the YB1 phosphorylation. To validate this conjecture, VSMCs were treated with MK2206, and the expression of pYB1 and CCL2 was both reduced in the MK2206-treated group compared with the control group detected by the Western blot (Figures 6A–D). Besides, pYB1 was also decreased in the MK2206-treated mice compared with the control group (Figures 6E,F). The *in vitro* and *in vivo* data indicate that targeting pYB1 could be a novel strategy for anti-atherosclerosis.

## Discussion

In the present study, we proposed pYB1 as a potential therapeutic target of atherosclerosis and demonstrated its molecular mechanism in atherosclerosis progress by regulating inflammation. We found that pYB1 expression was considerably elevated in atherosclerotic lesions in patients and mouse aortic arch. Gain- and loss-function assay *in vivo* confirmed that pYB1 played a key role in atherosclerosis by regulating inflammation, but not in lipid. Mechanistically, YB1 dephosphorylation increased the binding of GMD with CCL2 to promote *CCL2* mRNA decay. Combining this study and a preclinical study on the small molecular compound MK2206 (28), it is suggested that target YB1 phosphorylation will benefit atherosclerosis therapy by reducing inflammation.

Previous studies showed that monocytic YB1 expression is positively associated with pro-inflammatory cytokines infiltration of the vessel wall in hemodialysis patients (42, 43), while phosphorylation of YB1 at Ser102 (homo sapiens) in response to endotoxin, enhancing chemokine expression (44), and the expression of CCL5 is regulated by phosphorylation of YB1 at Ser102 in another study (45). The above researches suggest a crucial role of phosphorylation of YB1 at Ser102 in the regulation of pro-inflammatory cytokines, our study confirmed this speculation. In our study, we demonstrated that YB1 dephosphorylation at Ser100 (mus musculus) reduced mouse atherosclerotic plaque formation and plasma-CCL2 levels *in vivo*. Meanwhile, YB1 dephosphorylation decreased CCL2 by promoting mRNA decay in VSMCs *in vitro*. Besides, our previous study demonstrated that Akt-specific inhibitor MK2206, a clinical phase II drug, showed an anti-atherosclerosis activity *in vitro* and *in vivo*, and it reduced plasma-CCL2 levels in *ApoE*^−/−^ mice (28). Interestingly, as a downstream of AKT, pYB1 is downregulated in MK2206 treated-VSMCs and -mice, which were shown in this study. These results suggested that MK2206 attenuates atherosclerosis by inhibiting the phosphorylation of YB1. This further indicated the great potential for clinical application in attenuating atherosclerosis by targeting phosphorylation of YB1 at Ser100/102.

CCL family proteins are members of pro-inflammatory cytokines which are implicated in a wide range of diseases (46, 47) and particularly in vascular disorders such as ASCVD (48, 49). CCL2 has been implicated as a key agonist for monocyte recruitment, which enhances the occurrence and progression of atherosclerosis (50). In mice of diverse genetic backgrounds such as low-density lipoprotein receptor-deficient mice (*LDLR*^−/−^) and *ApoE*^−/−^, CCL2 deficiency reduces most of the atherosclerotic plaque formation and macrophage infiltration (36, 37). Moreover, CCL2 and its receptor CCR2 are important mediators of macrophage accumulation in atherosclerotic lesions, which are considered targets to inhibit the progression of atherosclerosis (51–55). In addition, a phase II clinical drug MLN1202 (CCR2-specific antibody) (56) has been reported to have a good effect on reducing C-reactive protein (CRP) levels in atherosclerotic patients. In our study, we demonstrated that *CCL2* mRNA expression was dramatically downregulated in response to YB1 dephosphorylation. Consistently, we also revealed that CCL families were substantially inhibited by downregulating YB1 phosphorylation in atherosclerosis, leading to the suppression of atherosclerotic plaque formation.

Macrophages, endothelial cells, and VSMCs are key cell types in the development of atherosclerosis, and YB1 was also mainly expressed in the three cell types that were exhibited in the human and mouse single-cell sequencing data. Previous studies showed that YB1 inhibited VSMC proliferation and promoted VSMC differentiation (57), and it also decreased lipid uptake and inflammation in ox-LDL-treated macrophages (58), suggesting a potential role of YB1 in preventing vascular diseases. The above studies suggest that YB1 could play different roles in different cell types. In our study, we mainly demonstrated that YB1 dephosphorylation attenuated atherosclerosis *in vivo*, and YB1 dephosphorylation decreased *CCLs* (*CCL2, CCL7, CCL11, CCL12*) expressions in VSMCs *in vitro*, which may be a critical determinant of the reduction in atherosclerotic plaques. Although our study showed that YB1 is a key regulator of atherosclerosis progression by affecting inflammation in VSMCs, we cannot deny that YB1 in endothelial cells and macrophages may also play roles in the development of atherosclerosis. The cell-type-specific deletion or overexpression mice will help us to further define the functions of YB1 in different cell types in the future. In addition, we evaluated the expression of YB1 in human and mouse arteries with three samples in each group, the sample size is a limitation of our study. However, the upregulation of YB1 have be further supported by public data from 32 human samples. Moreover, sample size does not affect the major finding of this study, which is about the increased expression of YB1 phosphorylation in atherosclerosis and the related mechanism of CCL2 decay.

## Conclusion

It is crucial to treat inflammation in atherosclerotic cardiovascular disease as emerging therapies to improve the prognosis (59). Here, we have uncovered a novel role of pYB1 in regulating the progress of pathological atherosclerosis. YB1 dephosphorylation decreased lipid deposition and inflammation *in vivo*. In addition, pYB1 deletion in VSMCs reduced CCL2 expression by promoting GMD-mediated mRNA decay *in vitro*. Our study highlights that YB1 dephosphorylation decreased inflammatory response and provides novel therapeutic targets for atherosclerosis.

## Data availability statement

The datasets presented in this study can be found in online repositories. The names of the repository/repositories and accession number(s) can be found below: https://doi.org/10.5281/zenodo.5785222, https://www.ncbi.nlm.nih.gov/geo/, GSE43292, https://www.ncbi.nlm.nih.gov/geo/, GSE120521, https://www.ncbi.nlm.nih.gov/geo/, GSE131780.

## Ethics statement

The studies involving human participants were reviewed and approved by Ethics Committee of Peking Union Medical College Hospital. The patients/participants provided their written informed consent to participate in this study. The animal study was reviewed and approved by Animal Care and Use Committee of Peking Union Medical College.

## Author contributions

JW, HZhan, and HZhao: conceptualized the study. JW conceived and supervised the project. HZhan, YT, and ZL prepared the manuscript. The method used in assigning the authorship order among co-first authors is the importance of each author's work. HZhao and HY performed most of the animal experiments. YT performed most of the molecular biological experiments, analyzed the data, and prepared the figures. ZL performed all the bioinformatics analysis in this study. CG, SL, YX, and YY assisted with the routine experiments. BL and TZ performed the lentivirus construction. All authors read and approved the final manuscript.

## Funding

This study was financially supported by the National Key Research and Development Program of China, Grant Nos. 2019YFA0801804 and 2019YFA0801703 (to JW), the National Natural Science Foundation of China, Grant No. 81800359 (to HZhao), and the Chinese Academy of Medical Sciences Innovation Fund for Medical Sciences, Grant No. 2021-I2M-1-016 (to HZhao).

## Conflict of interest

Author BL was employed by the company Jilin Zhongtai Biotechnology. The remaining authors declare that the research was conducted in the absence of any commercial or financial relationships that could be construed as a potential conflict of interest.

## Publisher's note

All claims expressed in this article are solely those of the authors and do not necessarily represent those of their affiliated organizations, or those of the publisher, the editors and the reviewers. Any product that may be evaluated in this article, or claim that may be made by its manufacturer, is not guaranteed or endorsed by the publisher.
