# Supplementary material for: YB1 dephosphorylation attenuates atherosclerosis by promoting CCL2 mRNA decay
Source: Front Cardiovasc Med. 2022 Aug 4;9:945557. doi: 10.3389/fcvm.2022.945557 (PMC9386362; doi:10.3389/fcvm.2022.945557)
Supplement: Supplementary file 1 [file Data_Sheet_1.docx]

Supplementary Material

**PUBLIC DATA ACCESS**

Data collected in the study will be made available using the data repository Zenodo (http://doi.org/10.5281/zenodo.5785222). The microarray data of the expression matrix for plaque and intact carotid tissues of atherosclerosis were retrieved from NCBI’s GEO dataset (GSE43292, https://www.ncbi.nlm.nih.gov/geo/query/acc.cgi?acc=GSE43292) ([1](#_ENREF_1)). RNA-seq data of human stable and unstable atherosclerotic plaques were retrieved from the GEO dataset (GSE120521, https://www.ncbi.nlm.nih.gov/geo/query/acc.cgi?acc=GSE120521) ([2](#_ENREF_2)). Single-cell sequencing data of atherosclerotic lesions in both human and mouse were retrieved from the GEO dataset (GSE131780, https://www.ncbi.nlm.nih.gov/geo/query/acc.cgi?acc=GSE131780) ([3](#_ENREF_3)).

**Reference:**

1. Ayari H, Bricca G. Identification of two genes potentially associated in iron-heme homeostasis in human carotid plaque using microarray analysis. *J Biosci* (2013) 38:311-5. doi: 10.1007/s12038-013-9310-2.

2. Mahmoud AD, Ballantyne MD, Miscianinov V, Pinel K, Hung J, Scanlon JP, et al. The Human-Specific and Smooth Muscle Cell-Enriched LncRNA SMILR Promotes Proliferation by Regulating Mitotic CENPF mRNA and Drives Cell-Cycle Progression Which Can Be Targeted to Limit Vascular Remodeling. *Circ Res* (2019) 125:535-51. doi: 10.1161/circresaha.119.314876.

3. Wirka RC, Wagh D, Paik DT. Atheroprotective roles of smooth muscle cell phenotypic modulation and the TCF21 disease gene as revealed by single-cell analysis. *Nature medicine* (2019) 25:1280-9. doi: 10.1038/s41591-019-0512-5.

**1 Supplementary Figures and Tables**

- 1. **Supplementary Figures**


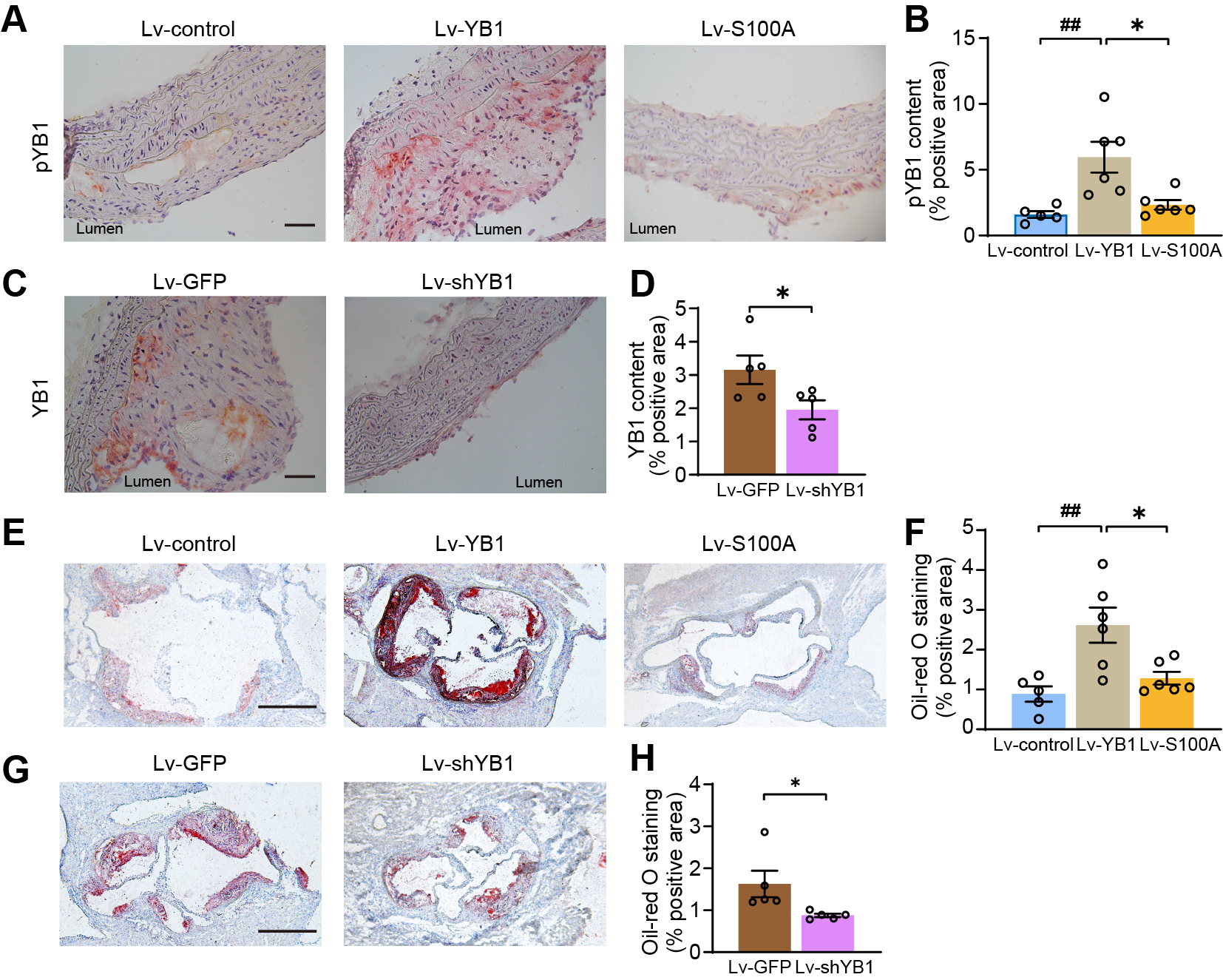


**Supplementary Figure 1:** Validation of the immunohistochemical changes of YB1 expression and YB1 dephosphorylation decreased lipid deposition in ApoE*^-/-^* mouse. **(A, B)**. Representative images of immunohistochemical staining of pYB1 in the mouse aortic arch from Lv-control, Lv-YB1, and Lv-S100A group, and the related quantitative analysis, respectively. **(C-D)** Representative images of immunohistochemical staining of YB1 in the mouse aortic arch from Lv-GFP and Lv-shYB1 group, and the related quantitative analysis, respectively. **(E-F)** Representative images of Oil Red O staining of the root from Lv-control, Lv-YB1, and Lv-S100A group, and the related quantitative analysis. **(G-H)** Representative images of Oil Red O staining of the root from Lv-GFP and Lv-shYB1 group, and the related quantitative analysis, respectively. ^##^*P* < 0.01 vs. Lv-control, **P* < 0.05 vs. Lv-YB1. Data are presented as the mean ± SEM. Statistics B, F: One-way ANOVA, D, H: Student’s 2-tailed t-test. Scale bar A, C= 25 μm, E, G = 500 μm. (n = 5 or 6 for each group).


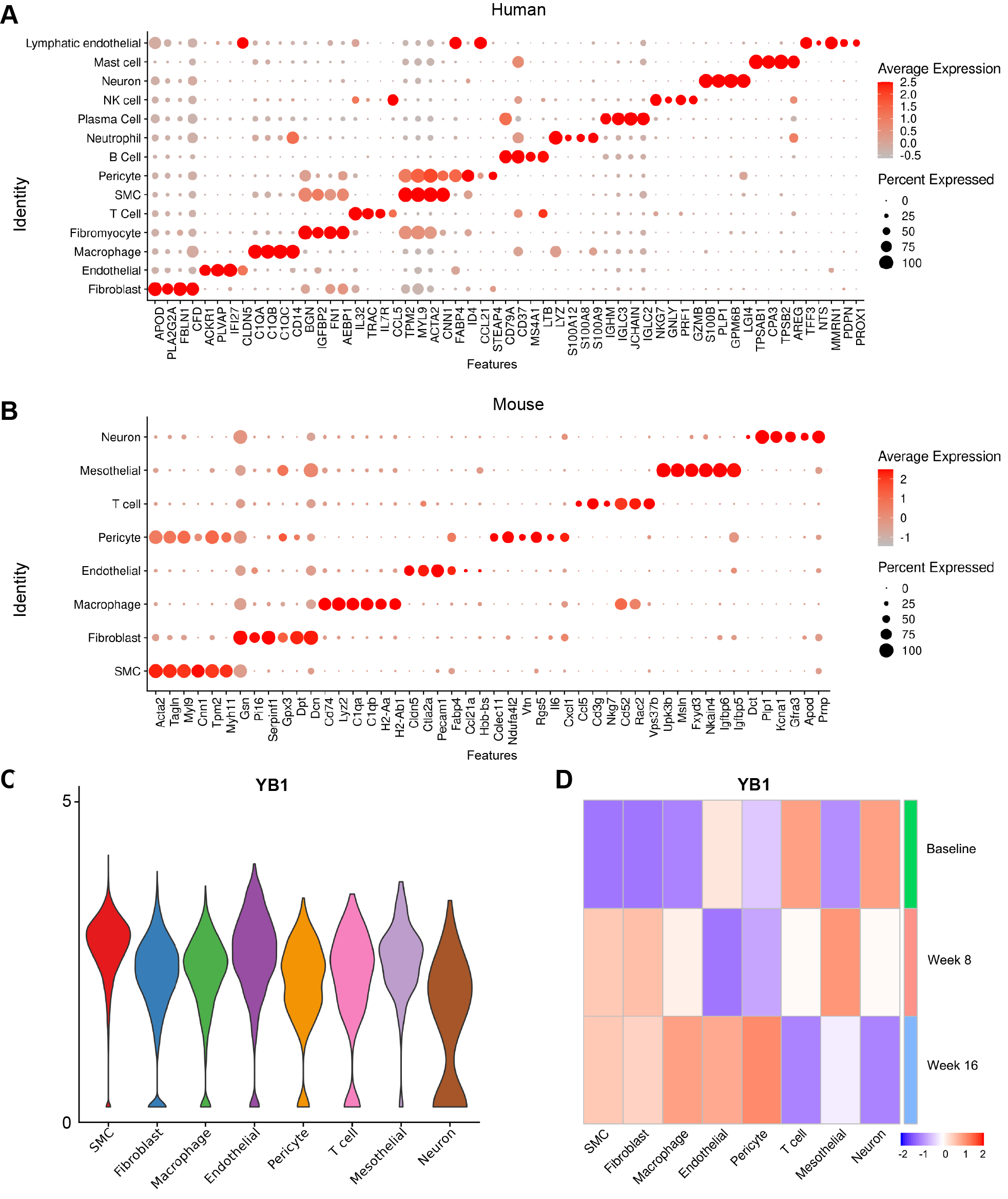


**Supplementary Figure 2:** Annotation of cell types in single-cell data. Dot plots representing expression levels of highly expressed genes defining each cell type in human **(A)** and mouse **(B)** data. **(C)** Violin plot visualizing the expression of YB1 in cells of mouse aorta. **(D)** Heatmap showing the relative expression levels of YB1 were increased in VSMCs, fibroblasts, and macrophages in the mouse aorta at the 8^th^ and 16^th^ weeks of atherosclerotic model compared to controls.


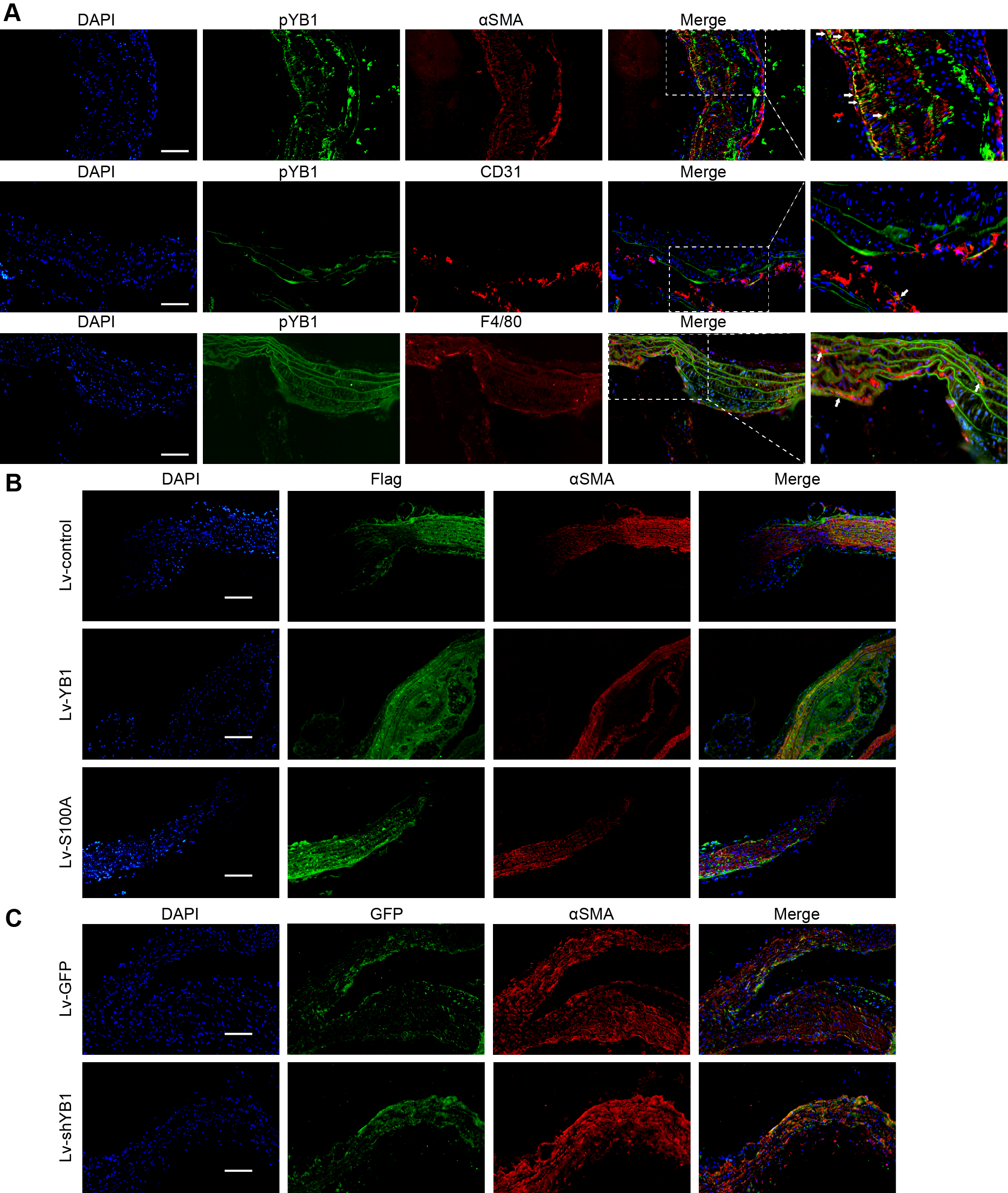


**Supplementary Figure 3:** pYB1 mainly localized in aortic smooth muscle cells. **(A)** Representative images of immunofluorescent co-staining of pYB1 and marker of smooth muscle cell (αSMA), endothelial cell (CD31), or macrophage (F4/80), respectively. **(B)** Representative images of immunofluorescent co-staining of αSMA and exogenous YB1 with Flag antibody. **(C)** Representative images of immunofluorescent co-staining of αSMA and GFP in aortic arches infected by lentivirus. Scale bar = 100 μm.


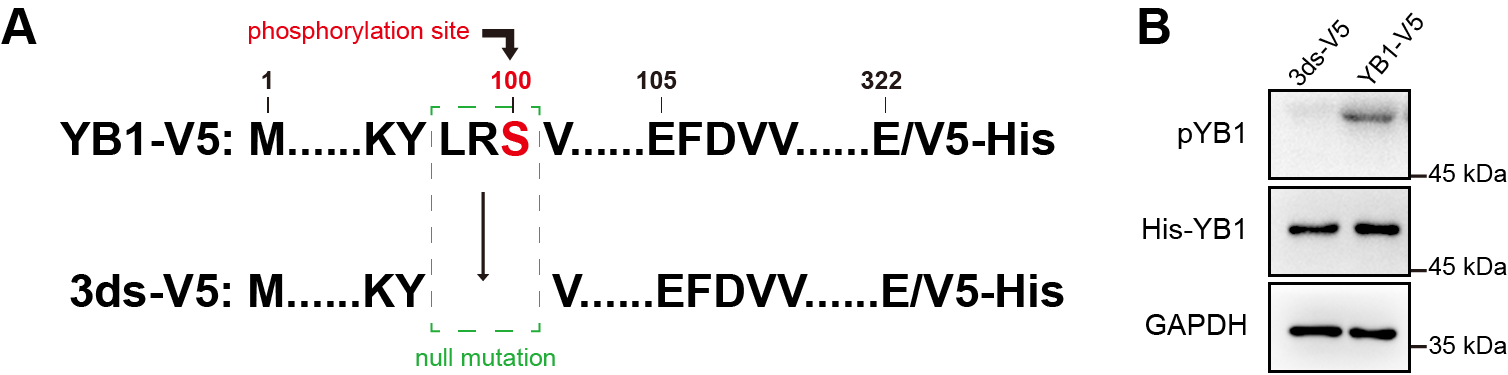


**Supplementary Figure 4:** Construction of YB1 serine phosphorylation site mutant smooth muscle cell line. **(A)** The amino acid sequence of wild-type protein YB1-V5 and the mutant protein 3ds-V5. **(B)** Validation of YB1 phosphorylation mutant smooth muscle cell line by western-blot.

**1.2 Supplementary Tables**

**Supplementary Table 1:** Antibodies for Western Blot

| Antibodies | Categories Number | Manufacturer | Dilution Ratio |
| --- | --- | --- | --- |
| GAPDH | 10494-1-AP | Proteintech | 1:5000 |
| Phospho-YB1 (Ser102) | 2900S | Cell Signaling Technology | 1:1000 |
| YB1 | Y0396 | Sigma Aldrich | 1:1000 |
| CCL2 | ab25124 | Abcam | 1:1000 |
| His | SAB1306082 | Sigma Aldrich | 1:3000 |
| Flag | F3165 | Sigma Aldrich | 1:5000 |
| DCP1A | D5444 | Sigma Aldrich | 1:1000 |
| GR | 12041S | Cell Signaling Technology | 1:1000 |
| UPF1 | 12040S | Cell Signaling Technology | 1:1000 |
| PNRC2 | ab235599 | Abcam | 1:500 |
| HRSP12 | PA5-31352 | Invitrogen | 1:500 |
| Goat Anti-Rabbit IgG H&L (HRP) | ZB-2301 | Zsbio | 1:5000 |
| Goat Anti-Mouse IgG H&L (HRP) | ZB-2305 | Zsbio | 1:5000 |

**Supplementary Table 2:** Antibodies for Immunohistochemical and immunofluorescent staining

| Antibodies | Categories Number | Manufacturer | Dilution Ratio |
| --- | --- | --- | --- |
| CCL2 | NBP2-22115 | Novus Biologicals | 1:200 |
| YB1 | Y0396 | Sigma Aldrich | 1:100 |
| Phospho-YB1 (Ser102) | 2900S | Cell Signaling Technology | 1:100 |
| αSMA | ab5694 | Abcam | 1:500 |
| αSMA-FITC | F3777 | Sigma Aldrich | 1:200 |
| F4/80 | ab6640 | Abcam | 1:100 |
| CD31 | 565629 | BD Bioscience | 1:200 |
| GFP | Ab13970 | Abcam | 1:200 |
| Flag | F3165 | Sigma Aldrich | 1:200 |
