# Supplementary material for: YB1 dephosphorylation attenuates atherosclerosis by promoting CCL2 mRNA decay
Source: Front Cardiovasc Med. 2022 Aug 4;9:945557. doi: 10.3389/fcvm.2022.945557 (PMC9386362; doi:10.3389/fcvm.2022.945557)

Supplementary Data 1. Original images of the Western blot analysis

Figure 5c-First

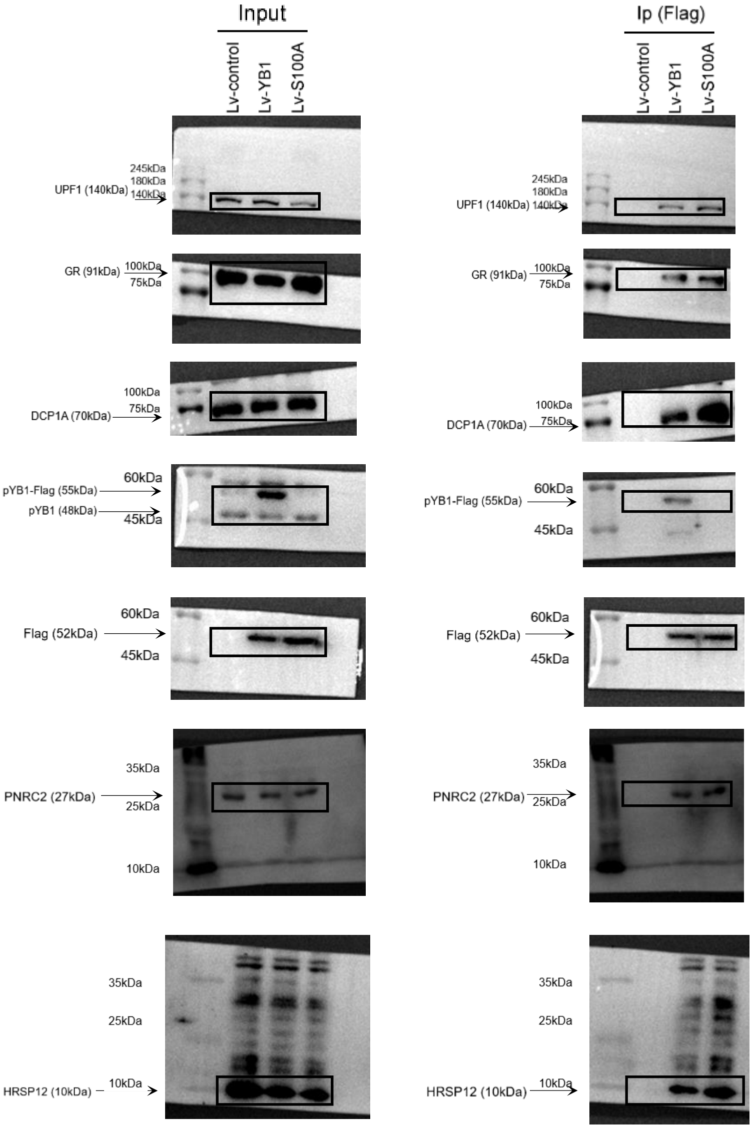

Figure 5c-Second

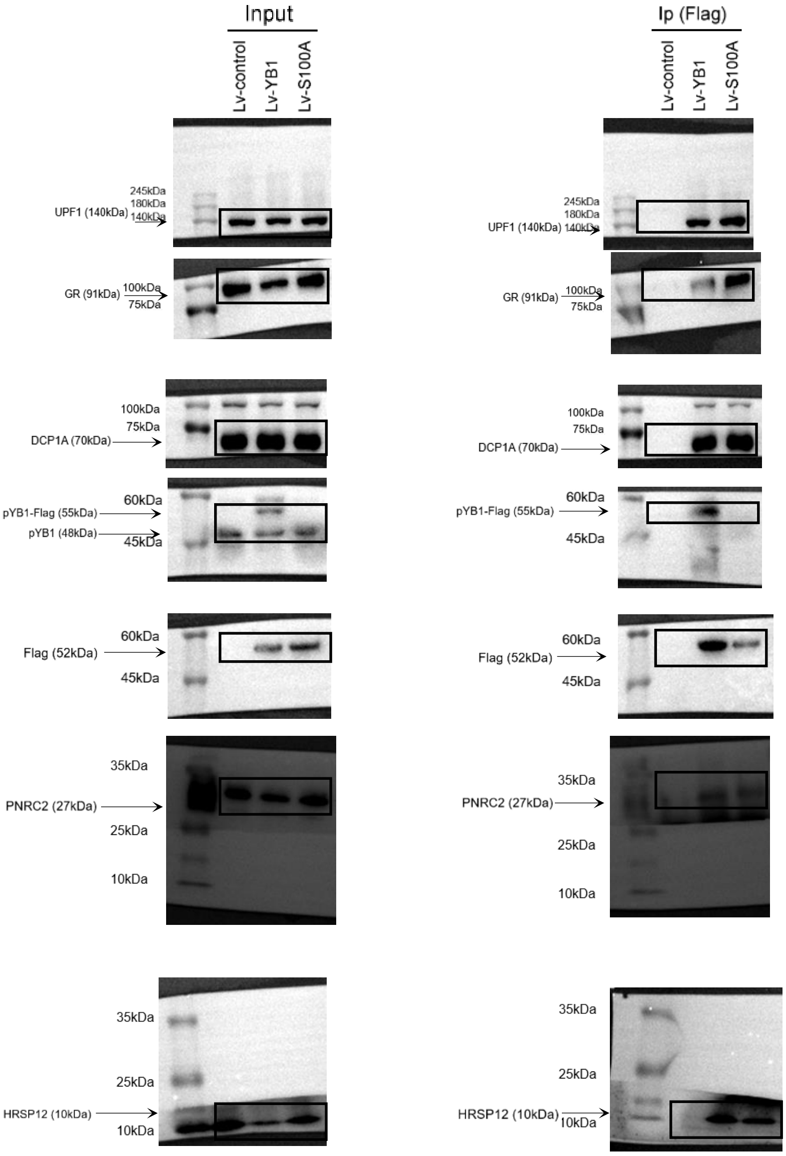

Figure 5c-Third

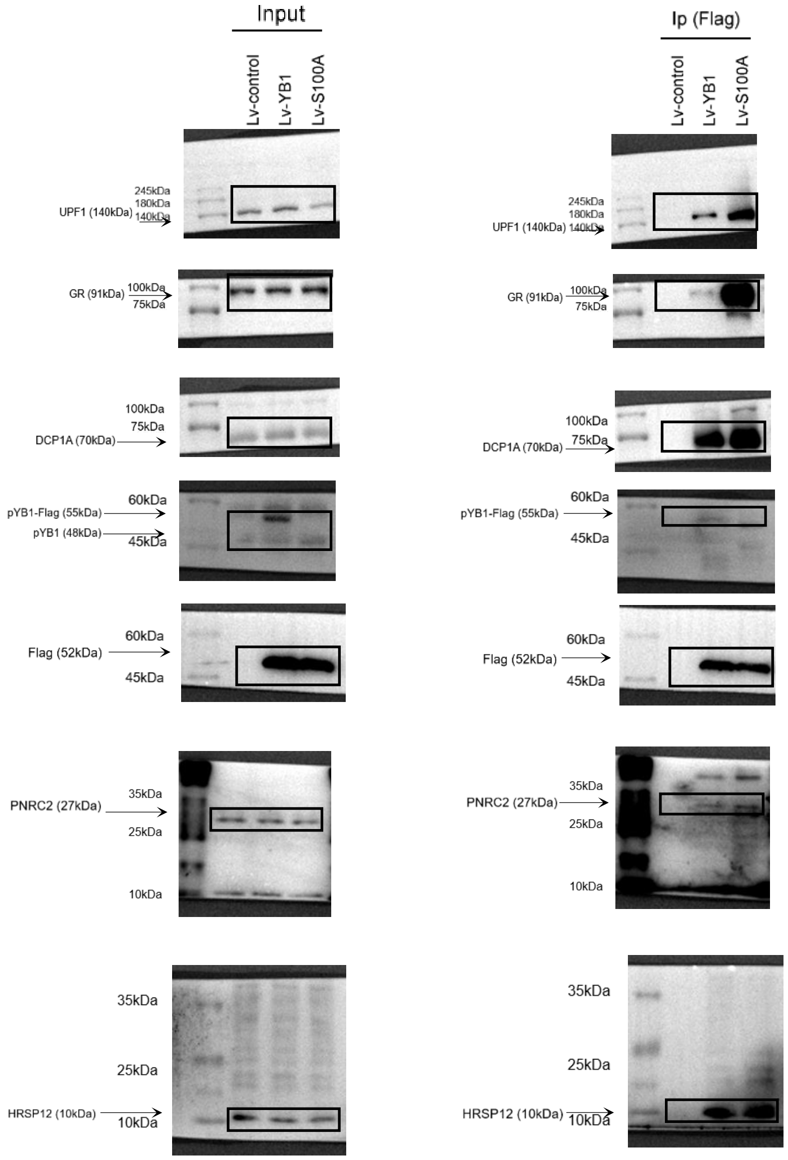

Figure6a

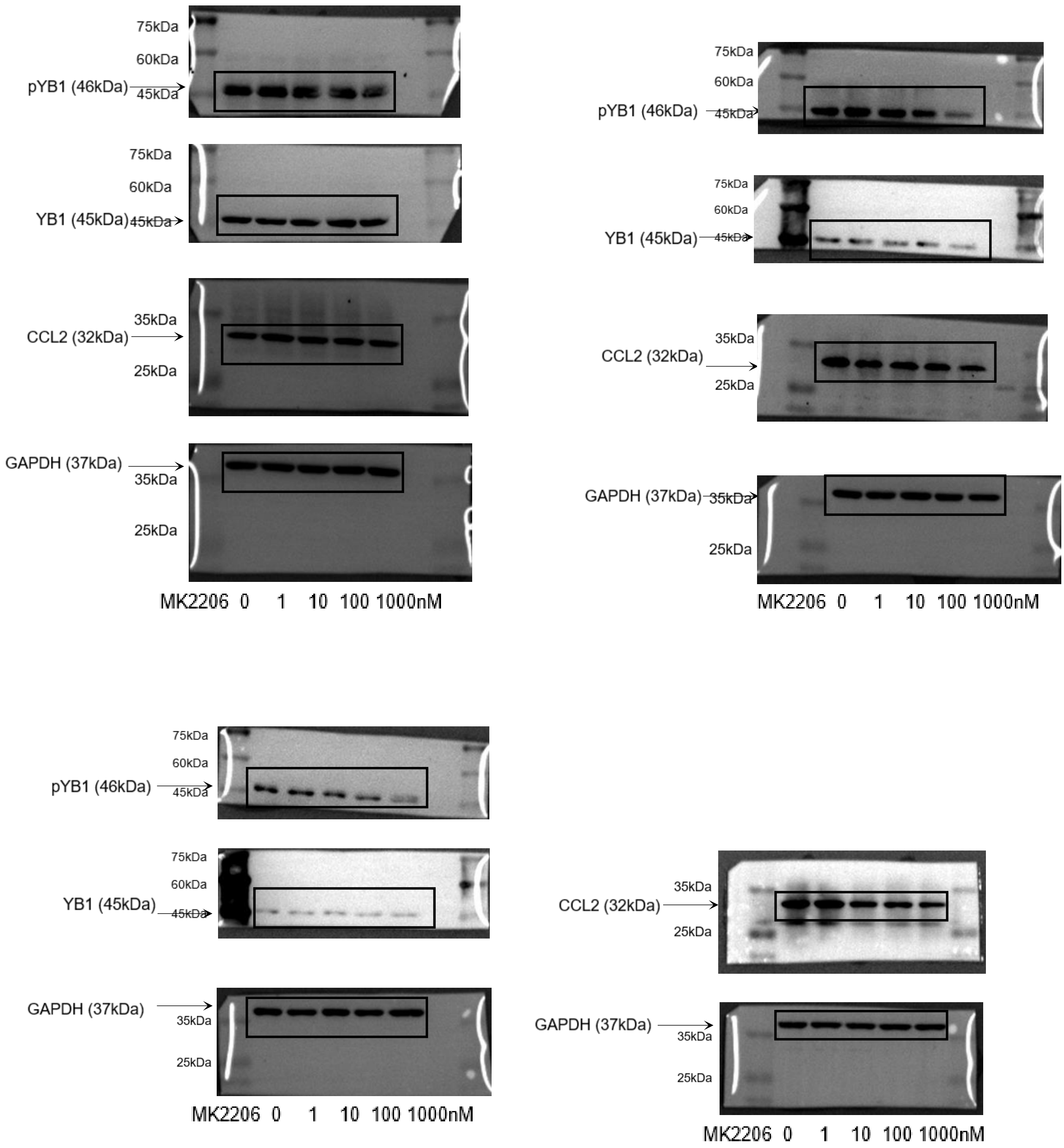

75kDa
60kDa
pYB1 (46kDa)
45kDa
YB1 (45kDa)
35kDa
CCL2 (32kDa)
25kDa
GAPDH (37kDa)
MK2206 0 1 10 100 1000nM

Supplementary Figure 4b

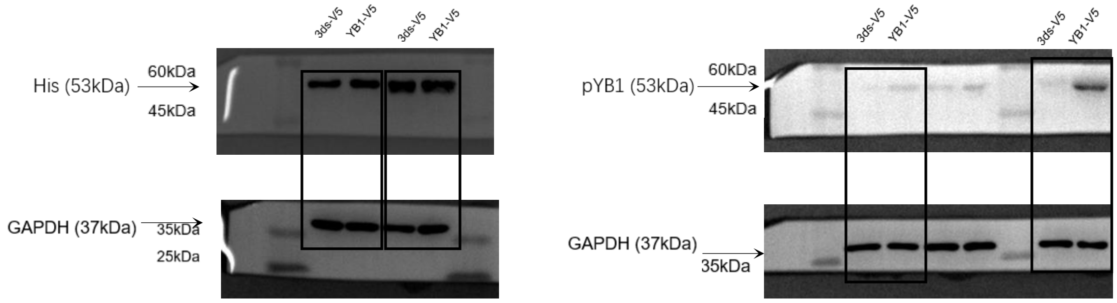

Supplement: Supplementary file 2 [file Data_Sheet_2.PDF]
